# Book Reviews

**Published:** 1807-06

**Authors:** 


### CRITICAL ANALYSIS

OF THE

### RECENT PUBLICATIONS

ON THE

### DIFFERENT BRANCHES OF PHYSIC, SURGERY, AND MEDICAL PHILOSOPHY.

---

*Observations on Morbid Poisons, Chronic and Acute. The First comprehending Syphilis, Yaws, Sivvens, Elephantiasis, and the Anomala confounded with them. The Second the Acute Contagions, particularly the Variolous and Vaccine. Second Edition, illustrated with Coloured Engravings, and further Commentaries on the Doctrines of Mr. Hunter.* By JOSEPH ADAMS, M. D. F. L. S. Physician to the Small-Pox and Inoculation Hospitals. Quarto. London, 1807.

As the first edition of this work was published before the commencement of our Journal, we may review the present as an original performance. To this, the length of time since the former edition has been out of print, and the copious additions to the present, might fairly entitle it.

On account of the novelty of some opinions, which the author professes to have derived from Mr. Hunter, he has thought it necessary to enter on a "preliminary discourse, to shew that the common mode of reasoning among medical writers, is *by no means satisfactory*, and very far removed from that plan of induction first proposed by Bacon, and since gradually introduced into the other sciences. He gives credit indeed to Sydhenham for accurate descriptions and excellent practical remarks; but urges, that the unfounded theories attached to them have proved injurious to his more solid precepts, and a means of leading his followers into an erroneous practice. Mr. Hunter, if we may believe our author, is the only medical writer who has asserted nothing which he could not prove, or whose theories are all supported by actual experiment or fair induction. Perhaps, in this zeal for his master, he may have taken more pains than are necessary to establish a reputation so generally admitted. He should recollect too, that Mr. Hunter flourished later, and consequently ought to be more correct than either of the great names to whom he is opposed; and, we conceive, that he would not consider himself much honoured by shewing his superiority over most of those writers, who have ventured to attack him during life, or after death. Even admitting the apology before introduced, for this part of his work it might certainly have been considerably shortened; we shall therefore only offer the spirit of it in as few words as possible.

We

We are first told, it was remarked by Mr. Hunter, that "no person has a correct idea in his own mind, unless he can communicate it to another," (we hardly suppose our author means to say, that Mr. Hunter was the first person who made this remark). It is afterwards added, that "such a position came with peculiar force from one, who always felt a difficulty in explaining himself in popular language, and was even accused by some of being ignorant of his own meaning. It is admitted, that Mr. Hunter showed often an awkwardness at expressing himself in popular language, but this is imputed to the deficiency or inaccuracy of that language; and it is said, that Sir Isaac Newton, and other original genius," found the same difficulty.

After this, our author, we suppose, to convince us of his impartiality, admits that Mr. Hunter's language has been improved by Mr. Abernethy. We have, before now, expressed our opinion of Mr- Abernethy's additional syllable to the word *morbid*,* nor do we see any thing in the argument of Dr. Adams to induce us to alter it. On the contrary, we object to the word "morbific" *in toto*, as having no meaning when coupled with poisons, since nothing can be a *poison* which is not *morbific*. We have dwelt longer on this subject than is necessary; first, because from Dr. Adams we expect a more than common accuracy in the choice of words, and next, because we think it fair to take every just opportunity of defending ourselves, when we presume to differ from such well received writers, as the two with whom we are at issue. The rest of these remarks refer to some passages in Messrs. Bell's, in which, they presume to treat Mr. Hunter with levity: an offence which seems, in the opinion of our author, nothing less than a high crime and misdemeanour, if not absolute treason. Dr. Beddoes is handled with more civility, inasmuch as his difference with Mr. H. is supposed to arise from inadvertency.

We have next a general analysis of the Baconian mode of reasoning by induction, after an accurate and experimental tracing of the series and order of every event. An historical account of the discovery of *hernia congenita* is produced, to shew the errors into which Haller and Pott fell for want of pursuing this plan, and of the satisfactory explanation of Mr. Hunter, in consequence of avoiding all inferences till he had traced every fact.

Mr. Gibson, is however, allowed the merit of completing one of Mr. Hunter's experiments by pursuing a similar train of induction. This is introduced to teach the reader, that though Mr. Hunter has marked out a path, which, whenever, like Bacon, he may desert, we are to recover, that is, to pursue the question as far as it is satisfactorily proved by experiment, before we venture to draw conclusions. In this manner, the author proceeds

* See our Review of Mr. Abernethy's last Work.

ceeds to complete two important inquiries, which Mr. Hunter is said to have left unfinished. The first is, concerning one supposed cause of impotence; the second, the experiments on the digestion of the stomach after death. The first must depend altogether on reasoning, but the second being a matter of experiment, we must do Dr. A. the justice to admit, that the whole has proved to us a most interesting recital of inferences fairly deducable from well marked facts. The experiment was made publickly in Mr. Brooke's Anatomical Theatre, on a rabbit whose stomach was digested after death by its own juices, and its contents found loose in the abdomen. From this, and the other circumstances attending the examinations, it appears, that Mr. Hunter, though apparently unable to account for the uncertainty of the digestion of that viscus, was right in the following very important doctrines.

That there are different modes in which animals die, and that these different modes are attended with different phænomena, all depending upon, and easily accounted for by, the manner of dying.

That the gastric juice is probably the same in all animals, whatever may be the nature of their food.

That nothing but the life of the stomach preserves it from being digested by its own secretion.

That the coagulation of the blood after apparent death, depends as much on the life of the blood, as the stiffening of the limbs does on the contraction of the muscles, and that both may be prevented by a similar mode of dying.

Our author having thus satisfactorily proved some of his masters most favourite and most disputed doctrines, might, we think, have been contented with his success, and have omitted the concluding triumph over two of Mr. Hunter's adversaries.

The necessity of conducting our experiments and inductions in the manner proposed by Bacon, and followed by Hunter, being thus considered as established; the subject of morbid poisons is entered upon by a definition of the terms endemic, epidemic, infection, and contagion, each of which is confined to distinct and determined meaning.

After this, we have a full explanation of Mr. Hunter's distinctions between susceptibility, disposition and action, and also a great number of illustrations of the doctrine that no two diseased actions can be maintained at the same time in the same constitution. This is proved from the writings of many well received authors, who were not aware of the inferences deducable from the facts stated by themselves; the few objections which have been offered are answered, and this part is concluded by a most important and interesting account of the manner in which the variolous and vaccine poisons are made to pursue their usual course, at the same time, sometimes without interruption to, and at others producing, apparently, a mutual effect on each other. All this is proved by Extracts from the Hospital Registers, of which we shall transcribe the second as the shortest.

"On the 21st November, 1805, Thomas Froome, aged 15 years, in service in London, was seized with natural small-pox. His employer inconsiderately sent him to his family, consisting of his parents and seven other children, neither of whom had gone through the disease. As they lived only a few miles from town, the father applied to the hospital for assistance. His son was received into the house, and the father advised to be vaccinated, with all his family, in order to supercede the variolous infection. When every argument proved ineffectual, the whole family was admitted into the inoculating house, and inoculated from Thomas. His eruptions were of the opake white sort, a distinction which has not been made either by Sydenham or Frend, but which, if there is a kind more favourable than others for inoculation, has hitherto claimed the preference. The character of the pustule was preserved in the whole family, the insertions proving more circumscribed than usual, and the whole process being attended with very little fever and few eruptions.

"From one of these a boy was inoculated, who had an hundred pustules, which retained the same character. He had but little fever, and the inoculated part was circumscribed.

"From him a subject was inoculated with three insertions, and at the same time vaccinated with two insertions in the other arm. All the insertions preserved their character in all their stages, the variolous being circumscribed as above: he was feverish for two days and had five white secondary variolous pustules.

"From the above one person was inoculated from the vaccine insertion and another from the pustules in the face. The arm of the first pursued the regular process till the 16th day, when the areola was arrested, and one hundred white circumscribed pustules appeared.

"In the other, who was inoculated from the pustules in the face, the insertion was vaccine in all its stages. The vesicle much larger than common, and dried of the dark brown colour. A few papillary eruptions appeared, but never maturated.

"It is unnecessary to continue any further account of this register. The above cases are sufficient to show such an analogy as might be expected between two poisons, one of which is sufficient to render the constitution no longer susceptible of the other. I shall only remark, that in all the cases of both registers, the secondary pustules were variolous, having the exact character of the white sort above described. The secondary cow-pox eruption, when they appear, are totally different from the primary, and also from any kind of small-pox pustules, excepting the crystalline. Their resemblance to these made me very sanguine in my expectations that by inoculation they would assume the vaccine character. But in this I have been constantly disappointed."

The

The chapter concludes with a few aphorisms and commentaries deducable from the preceding facts.

The 3d chapter contains, "inferences from what has been recorded by other writers, which may illustrate the doctrine of morbid poisons." In this, the author begins with remarking, that though some morbid poisons are traced with sufficient accuracy for all practical purposes, yet, the same cannot be said of the venereal and others confounded with it. After hinting at the hitherto uncertain descriptions given of yaws, sivvens, and a Canadian disease, he observes, that local complaints on the fauces and genitals, parts most frequently in contact with other subjects, were common long before the venereal disease was known. As a proof of this, he mentions the number of remedies for ulcers in these parts mentioned by Pliny, and the accuracy of Celsus in describing nine different kinds of ulcers to which the penis is liable. These he shows frequently occur at this time, and are often confounded with the venereal. Instances of each are given from various writers, who have described such ulcers on the genitals, on the female nipple, and in the fauces of sucking children ; on the lips and in the gums from the transplantation of teeth. All these, whenever they are accurately described, are shown to be different from the true venereal character, and reducable to one or other of those local diseases which Celsus describes in his chapter *de obscœnarum partium vitiis.*

In the next chapter, the author thinks it right to meet one difficulty, namely, If the genitals were always subject to so many local complaints, how it should happen, that the world was so much alarmed at the first appearance of syphilis ? This he imputes to the new complaint being incurable, till the free use of mercury was understood, to the greater certainty of its extension from the above cause, and from its existence in the gonorrhœal form, and also from the slow progress of the chancre, which for some time would not prevent the venereal intercourse. Some remarks follow, on the probable causes of those morbid poisons which occur occasionally, and cannot be traced from any certain source. This, it is observed, is particularly striking in all the tooth cases, the subjects from whom the teeth were extracted being all in apparent health, and some of them at an age, when they could produce the only evidence that can be demanded, that they had never been exposed to the venereal contagion.

In all these cases, it is remarked, that a disease has been excited by mutual contact, which not being discoverable in more than one party, can only be imputed to the stimulus which the apparently healthy secretions of one person excited in the other. Of such, we can therefore trace their origin, atnd, for the most part, the disease ceases with the person first infected. But it seems more difficult to account for the first source of those contagions, which are now found to be propagated *ad infinitum ;* and yet, which there is every reason to believe, have not existed more than a certain number of years. A conjecture is offered

 that that they may have been derived from the brute creation, and the cow-pox is brought as an illustration. As this was suggested in his former edition, published a few years before Dr. Jenner promulgated his grand discovery, our author conceives it his duty to explain, that all his knowledge of this disease was derived from a correspondence with Dr. Jenner.

The fifth chapter is "on the first local actions induced by morbid poisons." This begins by showing the common causes, and subsequent processes of ulceration and sloughing, which are afterwards applied to the laws by which morbid poisons are governed; the same laws are also applied to increased and altered secretion on secreting surfaces.

In the succeeding chapter, we have "an account of the mode of cure, original and remedial, and of the difference between primary and secondary local actions." The natural mode of cure in common ulcers, or after sloughs, is first explained, that is, the manner in which the lost substance it restored, and the subsequent cicatrization follows. In morbid poisons the process is said to be altogether different, the lost substance never being restored, but the part skinned over without that contraction which produces the cicatrix after common ulcers. To this difference the author imputes the rapid healing of chancres after their venereal disposition is destroyed, the depression left where the loss of substance is considerable, the pitting after the small-pox, and several other instances related by different authors in their descriptions of supposed venereal cases. It is not less remarkable, that where the secondary local actions of a morbid poison are different from the primary, this law is not preserved, but the loss of substance is restored by granulation, as in common ulcers. The cause of the advantages derived from inoculation of small-pox is next traced, and also of the constant fatality of the disease when it occurs to the fœtus. Both are explained by the same law, namely, that the whole danger of small-pox depends on the number of pustules which follow immediately on the eruptive fever. This was well noticed by Sydenham, who first marked the early appearance of pustules on the face; and, that on the number and character of them only, depended our whole prognostic. In inoculated small-pox, our author shows, that these early pustules are confined to the place of insertion which lessens the danger, and in the fœtus every part of the skin being similar, the pustules over the whole body probably appear at the same time; hence the greater danger, and, as it has proved, the constant fatality of small-pox at that age.

After a full view of those processes, which attend the spontaneous cures of morbid poisons, we have an account of such as never yield but to remedies. This leads to a long disquisition on the actions excited by mercury, and the manner in which they induce a cure of various diseases, particularly the venereal.

Having thus pursued his enquiries into the laws common to morbid poisons in general, the author begins by the application of them them to the venereal, as the most generally known, and from its slow progress affording the best opportunities of marking every pecularity.

He first enters into the question of the probable antiquity of the disease, which he decides in the negative, more by authorities from the writings of the licentious poets, than of the physicians. The next question is on the identity of gonorrhœal and chancrous virus; a controversy, which the author conceives, never could have continued after Mr. Hunter's experiment, had it been recollected that though gonorrhœa may arise from a venereal cause, yet, that a discharge from those parts has existed from the remotest antiquity; nor have we any means of distinguishing the various causes in either sex. That the same poison may produce two different effects, according to the nature of the parts stimulated, is proved by the variolous poison, from the saliva of a patient, under which, he inoculated the disease as successfully as from the contents of the pustules.

It has been urged by those who object to the identity of the chancrous and gonorrhœal virus, that the latter was not described as a symptom of the disease till half a century after it was known in every other form. For this Dr. Adams accounts in the manner we have just stated, and concludes this part of his argument with the following paragraph.

"This is enough to lessen our surprize that gonorrhœa should be unnoticed for some time after the appearance of the venereal disease. But so far is it from proving the two contagions are different, that the fairest inference we can draw is in favour of their identity. For if by this time the disease began to be so far understood, that secondary symptoms were found the consequence of primary ones in the genitals, it is most probable that the first suspicion of venereal gonorrhœa arose from the occurrence of such secondary appearances where no other primary symptoms could be traced. I mean not to produce this in support of an argument, which rests on the basis of experiment, but as an answer to what has been urged on the contrary side."

The next enquiry is, whether gonorrhœa ever arises from ulcers in the urethra? In this, after many compliments to Mr. Whately, who has lately revived that doctrine, the author scruples not to decide in the negative. It is admitted, however, that mercury may often be useful in venereal gonorrhœa. The chapter concludes with some cases, in which secondary symptoms appeared in a manner, which required great difficulty in resolving.

The treatment of gonorrhœa follows. After which, we have a very minute description of chancres, their various appearances under different circumstances, and their treatment. The appearances and treatment of buboes occupy much of the author's attention, particularly, the phagedenitic form when exctted by mercury. In describing secondary symptoms, much pains are taken to explain Mr. Hunter's discovery, that they cannot be always prevented by any mode of exhiting mercury, though they may always be cured.

 A chap-

A chapter follows on a disease, known only in Scotland, and there called sibbens; the imperfect accounts hitherto given of which, induced our author to make a journey as far as Dumfries, in order to ascertain its true character.

( To be continued. )

---

*Sketch of the Revolutions of Medical Science, and Views relating to its Reform.* By P. J. CABANIS, Member of the National Institute of France, of the Medical Society and School of Paris, of the American Philosophic Society of Philadelphia, &c. &c. Translated from the French, with Notes, by A. HENDERSON, M. D. 8vo. pp. 420. 1806.

Citizen CABANIS informs us, that the present Work is little more than an Introduction to a much larger, which he intended to have composed, respecting "the application of the analytical method to the study of medicine." His declining health prevented his perfecting his plan; and if the whole was intended to take in as extensive a range as the Introduction announces, we are not surprised that the attempt was abandoned. What is now offered is, however, a truly valuable performance; the translation is not unworthy of the original, and is also enriched with many interesting additions, and some necessary corrections. In a few observations on the "design of the work," the Author takes occasion to remark the necessary changes which must occur in every science, in proportion as any fundamental truth is discovered. If such a discovery is not inconsistent with the floating theory of the day, it must in some measure strengthen it; but if it is incompatible with doctrines long admitted, its validity will be suspected; and when confirmed, must introduce new arrangements, and perhaps a considerable change in the language of the science.

It will not be disputed, that if there is a science more overcharged than another with "superfluous baggage," it is Medicine. "Influenced (continues our Author) by these powerful considerations, I had ventured to conceive the plan of a new classification of the different branches of medical research. I judged it advisable to adopt a new arrangement of the facts on which it rests, and of the particular ideas or opinions which their careful examination suggests; and without presuming to change its phraseology or its nomenclature, I hoped to be able, by rigorously determining the meaning of the words I employed, to banish entirely from its language, that vagueness and that obscurity which so very much disfigure it. This appeared to me a duty so much the more indispensable, that these defects may mislead even the most enlightened inquirers; and, above all, by furnishing the ignorant quack with an almost inaccessible asylum, they become, first, the source of the most fatal errors, and, afterwards, sanction them by a sort of mysterious attraction. As I proposed to consider the science of medicine, more particularly as it relates to the treatment of diseases, eases, it was to that branch of it which bears the name of Therapeutics, that all the others were to have been subordinate; it was in relation to it that all their subdivisions were to have been traced, and all their mutual connections determined; and the conclusions resulting from this new method of regarding things were all to have had the common aim of improving the practice of the art."

We are not surprised if M. Cabanis soon discovered that neither his strength nor time of life would permit him to indulge the hope of accomplishing such extensive objects. At the age when men begin to doubt the truth of their early decisions, or of what they have learned from their teachers, they first see the necessity of completely examining the source of a number of theories. Their own favorite opinions are so frequently contradicted by occurring facts, that before they can establish any thing satisfactory they too often give way to a general scepticism, or are alarmed at the magnitude of their own undertaking. Under such discouragements it may be asked, how are we to proceed in the important business of reform? The answer is obvious. We are carefully to collect such facts as are undisputed, and to cancel those opinions which are unsupported. Where a theory is insufficient for want of further experiment, we are to institute such experiment ourselves, or to call upon the author to do it. Where language is not sufficiently clear, and is made use of to prove by logic what can only be ascertained by fact, we are to mark the fallacy in others, and avoid a similar error ourselves.

When the great business of reform is thus simplified, all may join in producing it, and we may hope it will become progressive. But if before we begin, it should be thought necessary to show the source of error in our remote as well as nearer predecessors, and even our contemporaries, the work must be entered upon by those who can scarcely hope to see its accomplishment. Our readers will not suspect by this, that we wish to undervalue M. Cabani's labours; on the contrary, we are highly sensible of their value; what he has done will show us much of what has been attempted by others, and instruct us to avoid similar errors. In short, it will confirm what we shonld always have in view, that in our inquiries into Physiology, we should divest ourselves of every consideration but those laws by which we find life supported, and in Pathology should attend only to the deviations from such laws, and the means by which they may be restored.

The "View of the Revolutions of Medical Science, from its Origin to the present time," is so very cursory, that any attempt to abridge it would be unpracticable; we must therefore satisfy ourselves with giving the general contents, and offering such extracts as may show the reader the manner in which the whole is conducted. The various sections under this head comprehend — The cultivation of medicine by the chiefs of the savage tribes, by the poets, and principally by the priests.—Cultivation of medicine

 by by the first philosophers.—Of Hippocrates and the school of Cos. —Of the other schools of Greece.—From the time of the introduction of medicine in Rome to the epoch of the Arabians.—Epoch of the Arabians.—Introduction of medicine into Europe from Greece, with men of learning and of literature.—Of the Jewish physicians.—Of the first sect of chemical physicians.—Revival of learning and the Hippocratic system of medicine.—Of Stahl and Van Helmont.—Of Sydenham.—Discovery of the circulation of the blood.—Of Boerhaave.—Of Hoffman and Baglivi. —Of the new solidists of Edinburgh; and of the school of Montpelier.—Of the present state of medical education.——We shall select the description of Sydenham, and the improvements he introduced, not only as peculiarly interesting to our English readers, but as a specimen of the candour with which a French writer can sometimes treat English philosophy.

"When Sydenham appeared in England, the science of physic still retained its scholastic form. The progress of the other branches of knowledge had hitherto exerted only a prejudicial influence upon it; and the genuine spirit of observation was almost entirely unknown. Sydenham, after a short course of study, assisted by a little reading, but guided chiefly by the impulse of a happy genius, undertook to bring back the practice of the art to the path of experience. With the prevailing theories of the time he was but imperfectly acquainted; but this circumstance was, perhaps, more favourable to his labours, as it could never be embarrassing to his self-love, and as he would find the less difficulty in following the footsteps of Nature. Among the number of his friends was the illustrious Locke, to whom we are indebted, if not for the first principles of a philosophical method of inquiry, at least, for the first demonstration of the fundamental truths on which they are founded. The friendship of such a man sufficiently indicates the disposition of mind of the person who cultivates it, and serves, as it were, for its standard of comparison. We can, therefore, scarcely doubt, that the counsels of the philosopher must have greatly contributed to the success of the physician, who, indeed, acknowledges it himself with candour.

"Sydenham attacked, with the irresistible arms of experience, several destructive prejudices, which at that time prevailed. The chemists, for instance, had introduced into medicine the indiscriminate use of cordials, and of ardent or volatile spirits. In acute diseases, in particular, the abuse of these remedies was very great. Sydenham proved, that in such cases, they were almost always injurious; but especially at the commencement of the disorder. The small-pox, and other acute cutaneous eruptions were treated by sudorifics alone. Sydenham demonstrated, that this mode of practice had been more fatal to mankind, than a long succession of destructive wars. His *Treatise on the Gout* has been generally regarded as a master-piece of description: it is, indeed, the most perfect account of this disease which we possess; not, that this malady malady always presents itself in the manner in which it is described, but because we can conceive nothing more accurate or ingenious, than the plan of observation which he there lays down.

"Hippocrates, in his Epidemics, had sketched the outlines of a system of physic, as extensive as it was original: (I allude to his treatment of epidemical disorders). During several ages, his ideas had remained, in a manner, dormant. Baillou, a Parisian professor, in the 16th century, appropriated them to himself, and extended them; not, indeed, as a man of genius, for he was not such, but, at least, as an attentive observer and skilful practitioner. He was even led to consider them in several new points of view.

"Sydenham, without having any knowledge of Baillou, perhaps, even without having read Hippocrates, was led into the same path by observation alone. He pursued it with still greater success; and in this his chief glory consists. It is only since his time, that we have become thoroughly acquainted with those general variations to which the characters of epidemic diseases are liable; with the relations they bear to each other, and their connection with the different apparent changes of the atmosphere, or their independence of these changes, which is often very apparent; with the influence they exert upon sporadic or local disorders; and, lastly, with the manner in which their succession is regulated, although the order of it, we must confess, has not yet been subjected to any determinate rules, upon which we can entirely rely.

"The practice of Sydenham effected a real revolution in physic. It was the triumph, not of a transcendent genius, who reforms every thing by bold and general views, but that of an observer, who investigates with sagacity, who conducts his researches with skill, and who is always guided by a sure method. The theories of Sydenham were, it must be acknowledged, contracted, or even erroneous; and beyond the sphere of his experience, in which his natural penetration supplied the place of all other talents, his ideas were, in general, very limited; but no physician ever exerted so beneficial an influence on that branch of the art, to which all others are subservient—on its practical application: and in this respect, no one was ever more deserving of the title of restorer of true medical science."

In the third Chapter are comprised "General Views on the Subject of Medical Education." In this the Author begins by a short view of the faculties of man, the sources of his errors, and the invention of philosophy. This leads him to the analytical method, its difficulties, and still more, the difficulty of forming any classification of remedies. The necessity of system, and the danger with which it is often attended; of the influence of language on the sciences, particularly in medicine, so great a part of which has been hitherto necessarily hypothetical. This Chapter concludes with a section on the application of the analytical method to the business of Medical Education,

The fourth Chapter is on the various branches of medical research; and the fifth, on the accessory branches of study; a concluding summary follows, from which we extract this short energetic passage.

"The present period is one of those great epochs of history, towards which posterity will often look back, and of which it will expect a just account from those who had it in their power to accelerate and confirm the progress of the human mind, in the paths of amelioration. It is the lot of only a small number of fortunate individuals to exercise this extensive influence: but in the present advanced state of the arts and sciences, there is no one who may not, in some measure, contribute to their progress. The least real improvement in the most obscure art is quickly extended to all the rest, and the relations which have been established between the different objects of our labours, enable them all to benefit by the progress of each in particular. The ancients had, no doubt, a distant idea of these relations, and had perceived, that all the arts and sciences were connected together, and formed, as it were, a complete whole: but they had remarked this, without perceiving it distinctly, and had attempted to describe it, without knowing it thoroughly. It is only in recent times; it is only after having considered the various efforts of human industry in all their applications, and in all the different directions which they may assume; it is only after having subjected them to rules, and reduced them to common forms, that we have been enabled to determine with accuracy the mutual relations which connect them, and the influence which they exert, or are capable of exerting upon one another. We now know, and can demonstrate, that there is nothing insulated in the labours of man: they are united, if we may borrow the comparison, as nations are by their commercial ties: they mutually assits each other, like the members of the social community.

"The most obscure individuals, therefore, have it now in their power to render themselves eminently useful; men of science and letters, to whatever branches of research they devote their attention, artists, and even the meanest mechanics, all in their particular spheres, may now promote the general good, and contribute to the progressive improvement of the human race.

"And shall we then, who, devoting ourselves to the alleviation of the sufferings of mankind, so frequently command the interests that are dearest to the heart; we, whom the high importance of these interests obliges us to search for information in all quarters, and whose studies embrace almost all the branches of physical and moral research;—shall we alone be exempted from the duty of promoting the general welfare of mankind by our labours, and of contributing to their amelioration? Undoubtedly not. Let us, therefore, unite our efforts, and endeavour to introduce into the study and practice of our art, that superior reason and philosophy, without which so far is it from affording useful aid, that it becomes becomes a real public scourge: let us venture to connect it by new relations with the other branches of human knowledge; so that the latter, by its means, may be improved and illustrated. And at a time when the French Nation is about to consolidate its existence as a republic, let medicine, restored to its native dignity, commence a new career, which may prove rich in glory, and fruitful in benefits to mankind."

Some Notes are added by the Translator, which do credit to his accuracy and diligence.

